# Early Correction of Malocclusion Using Planas Direct Tracks

**DOI:** 10.1155/2013/395784

**Published:** 2013-09-03

**Authors:** Renata Reis dos Santos, Artênio José Isper Garbin, Cléa Adas Saliba Garbin

**Affiliations:** ^1^Universidade Estadual Paulista, UNESP Araçatuba Dental School, Preventive and Social Dentistry, Rua José Bonifácio 1193, Vila Mendonça, Araçatuba16015-050, SP, Brazil; ^2^Universidade Estadual Paulista, UNESP Araçatuba Dental School, Post-Graduation Program, Rua José Bonifácio 1193, Vila Mendonça, Araçatuba 16018-050, SP, Brazil; ^3^Universidade Estadual Paulista, UNESP Araçatuba Dental School, Orthodontics and Preventive and Social Dentistry, Rua José Bonifácio, 1193, Vila Mendonça, Araçatuba-16015-050, SP, Brazil

## Abstract

The correction of functional posterior crossbite through Planas Direct Tracks has many characteristics that can become advantages. The aim of this study was to present a clinical case showing how to use this procedure for early correction with resources available through public health services. The patient, a 4-year-old girl, arrived to receive treatment due to a functional unilateral crossbite. When the mandible was moved to the centric position, it was observed that the teeth had occlusal trauma. An occlusal adjustment was performed. The adjustment was not sufficient to promote functional equilibrium; thus, Planas Direct Tracks were made, resulting in functional equilibrium and correction of the malocclusion. As shown in the case report, the Planas Direct Tracks were effective for the correction of the posterior crossbite. If malocclusion is considered a public health problem, implementation of low-cost and easy-to-execute techniques is needed.

## 1. Introduction

Neuroocclusal rehabilitation (NOR) can be defined as a subfield of dentistry that studies the etiology of functional and morphological alterations of the stomatognathic system. Its aim is to investigate and eliminate etiological factors and avert the potential for lesions through selective occlusal griding and/or using Planas Direct Tracks. This innovation can be used at any stage of life to correct a crossbite [1].

The crossbite is one of the most common occlusal alterations. It is an abnormal relationship between the buccal or lingual surfaces of the superior teeth and the inferior teeth when the arches are in centric relation and may be unilateral or bilateral. The most common presentation is unilateral with a functional deviation of the mandibula crossed to the side. It can be classified as a dental crossbite, involving only one or more teeth, muscular or functional, in which case there is a possibility of adapting soft tissues through dental interference, or as a skeletal crossbite, in which alterations in the bone development cause asymmetric growth of the maxillaries [2].

Its etiology is a combination of many factors, including skeletal and neuromuscular components; however, the most frequent cause is palatal reduction [3, 4]. 

Self-correction is rare, and it is believed that early diagnosis and treatment of a posterior crossbite can help to avoid bone base dystrophies with orthopedic or structural alterations [5]. 

Several treatments have been applied for correction of these problems, and the studies converge on a common point: the therapy is used for deciduous dentition [6, 7].

 The Planas Direct Tracks technique is a component of many other neuroocclusal rehabilitation treatments and was developed by Pedro Planas. It constitutes an effective way to correct dental or functional alterations in children during deciduous dentition and the beginning of mixed dentition, normalizing dental occlusion, mandibular posture, condylar position, and masticatory function [5].

It is recommended as a first measure to facilitate early treatment of posterior crossbite, producing wear on the occlusals to remove the occlusal interference. In cases in which the occlusal wear is not sufficient to eliminate the interferences, the construction of tracks with photopolymerizable resin is suggested [1].

The aim of this study is to present a case report demonstrating how to execute the technique for early correction of crossbite with available resources in a dental public health service facility. 

## 2. Description of Case

**Figure 1:**
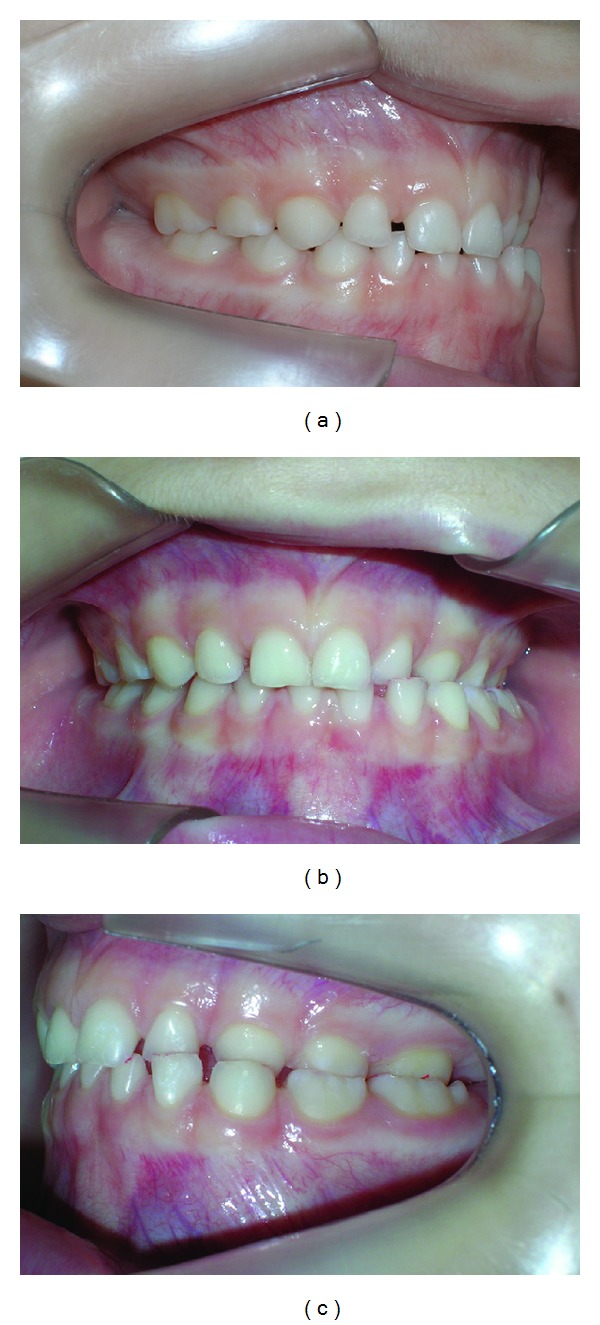
Clinical aspects in centric position. (a) Right side, (b) frontal view, (c) left side.

 The patient, JBC, a girl of 4 years and 5 months of age, arrived in search of treatment and showed a functional unilateral posterior crossbite on the left side (Figure 1), no caries lesions, normal soft tissues, and completed deciduous dentition. The crossbite was classified as functional because after mandibular manipulation to the centric position, it was possible to observe premature occlusal contacts. 

In this case, it was possible to verify a transversal decrease of the superior arch, which causes a condition of end-to-end biting of the canine teeth and occasional involvement of posterior teeth. In order to avoid discomfort, the patient moved the mandible to one side and then the other side until finding a position that allowed better adaptation, consequently resulting in the functional unilateral posterior crossbite. 

**Figure 2:**
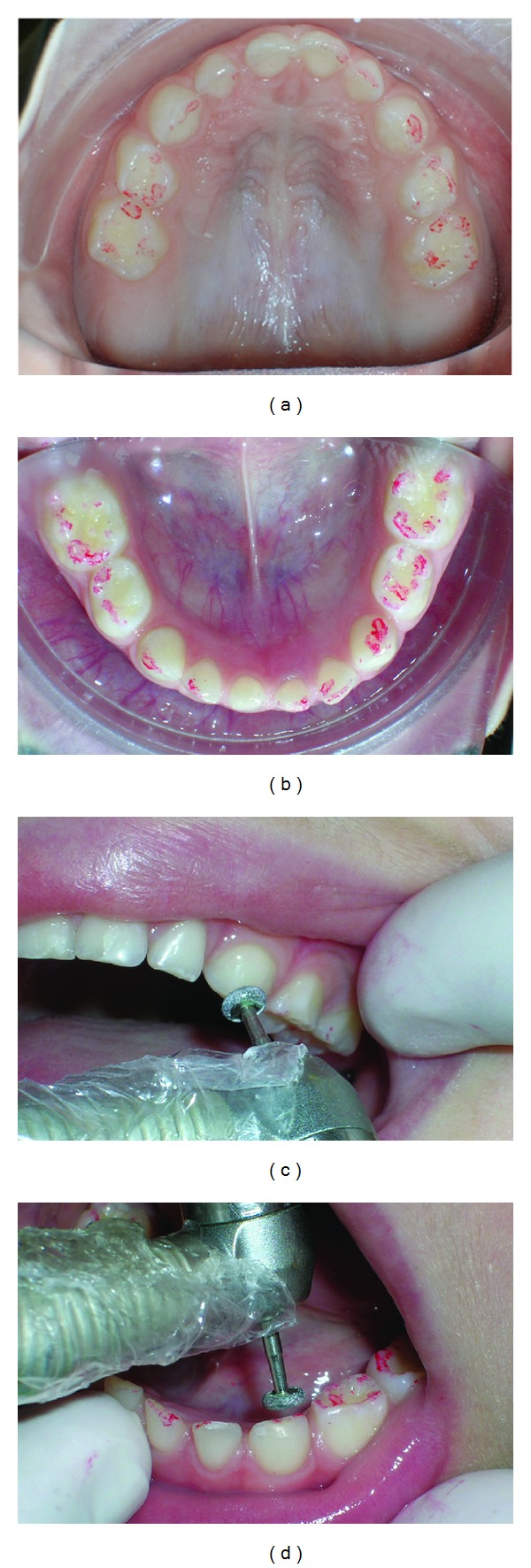
Oclusal view with carbon print and selective wears. (a) Superior oclusal view, (b) inferior oclusal view, (c) selective wears on superior teeth, and (d) selective wears o inferior teeth.

Guiding the mandible to the centric position, it was possible to observe that the teeth showed trauma, contributing to mandibular deviation and thus necessitating selective wears. A diamond bur number 3053 with a wheel form was used for high rotation with irrigation (Figure 2). 

**Figure 3:**
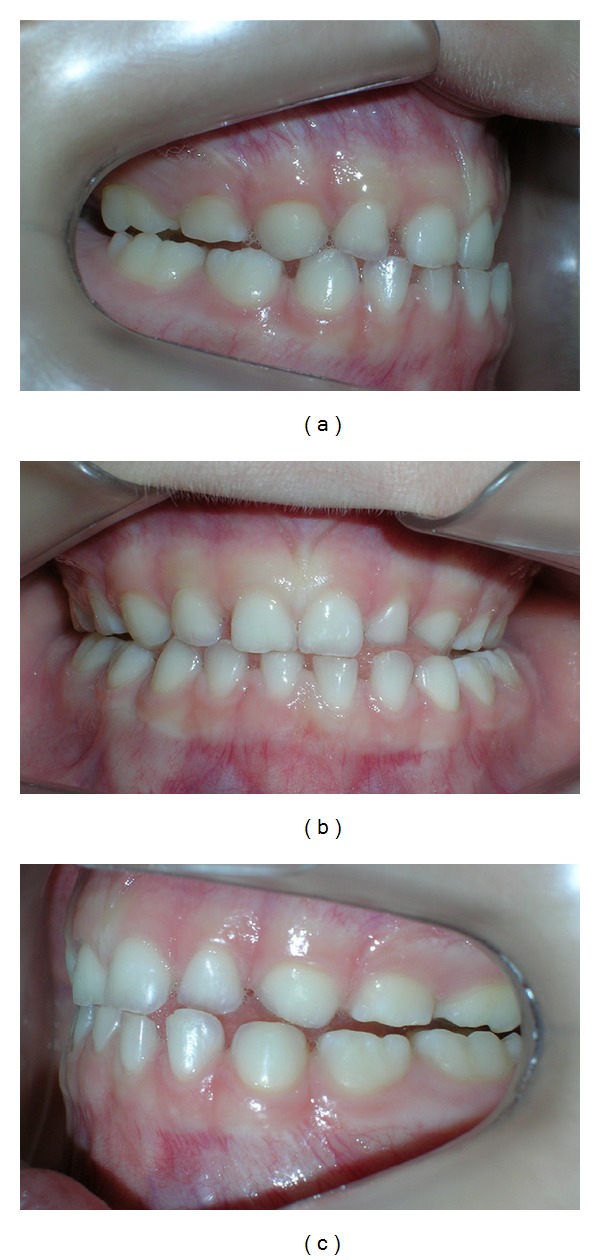
Clinical aspects after oclusal adjusts. (a) Right side, (b) front view, and (c) left side.

Due the fact that the adjustments were not sufficient to promote the equilibrium to allow the centralization of the median line (Figure 3), Planas Direct Tracks were made on the teeth involved in the crossbite (lateral incisive, canine, and left molars). 

**Figure 4:**
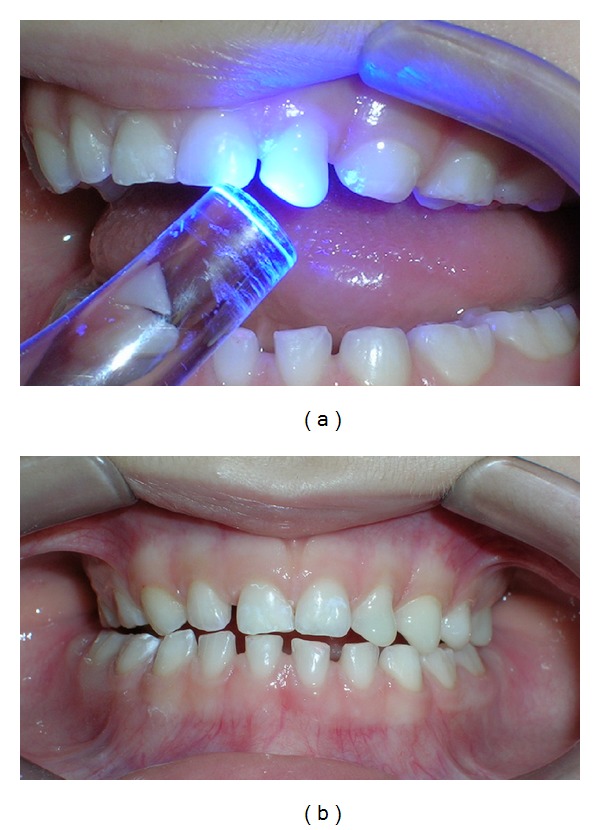
Confection of Planas Direct Tracks. (a) Photopolymerization of resin. (b) Front view after confection of Planas Direct Tracks centralizing the median line.

The standard procedure was followed using compost resin, conditioning with phosphoric acid at 37% for a duration of 30 seconds, washing and drying, and applying of adhesive and photopolymerized composite resin (Figure 4). The finishing was performed using diamond bur model 1192F and the Enhance bur. 

The extension of tracks should be large sufficiently to prevent the mandible from moving back to the comfortable position and should be thick enough to prevent fracture during function. It is suggested that occlusal wears should be made with a 45° angle in the tooth's vertical axis or at an angle greater than 30° to produce inclined planes that allow the mandible to attain an adequate expansion or force during mandibular closing [8].

After application of Planas Direct Tracks, new grinds were made. After treatment, the median line deviation was corrected and there was rehabilitation of functional equilibrium and correction of the malocclusion. 

**Figure 5:**
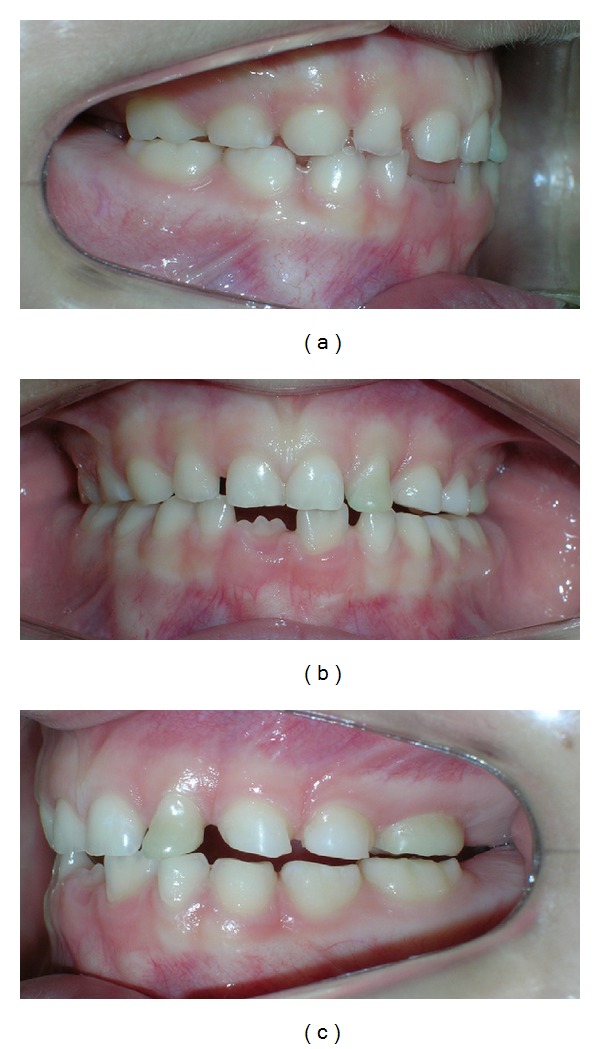
Clinical aspects after six months of treatment. (a) right side, (b) front view and (c) left side.

After 6 months, the tracks were evaluated again and reconstruction in one tooth and a new occlusal adjustment were found to be necessary (Figure 5). 

## 3. Discussion

Treatments using conventional braces were not effective for correction of the crossbite and did not modify the mandibular movements, probably due to the permanence of occlusal intereferences [9].

 Among treatment methods for posterior crossbite, the use of NOR, which includes the use of selective wears and Planas Direct Tracks, is efficient for correction of this anomaly [10]. These procedures are low cost, allowing the treatment of some malocclusions in public health service facilities. 

The cases indicated the occlusal adjustments through selective wears show unilateral crossbite, median line deviation, and occlusal interferences in deciduous dentition.

In a longitudinal study conducted by Lindner [11] to evaluate the early unilateral posterior crossbite using selective wears, the 76 children who were evaluated were divided in two groups, treated and untreated. In the untreated group, only 17% showed spontaneous correction of crossbite. Thus, early correction using selective wears is indicated to have a favorable prognosis.

The clinical report showed that the treatment started when the children were 4 years old because early intervention during the deciduous dentition phase may allow avoidance of the formation of several strucutural deformities in permanent dentition. Early diagnosis and correction, together with the physiological positioning of condyles and muscle equilibrium [1], prevent facial asymmetries in the adult phase [10]. The occlusal adjustment makes rehabilitation of neuromuscular activity possible, preventing masticatory movements during the growing phase. This treatment modifies the standard masticatory cycle, inducing bilateral chewing.

It is important to highlight that during the clinical exam for differential diagnosis between functional or true crossbite, mandibular manipulation should be performed and adjusted to the centric position. Functional crossbites show the meeting of median lines when mandibles stay in the centric position, which demonstrates the presence of occlusal interferences [8, 12]. 

The inclination of tracks should be made based on the characteristics of each case and should be related to Camper's plane and guide, allowing mandibular movements.

The major advantadge of Planas Direct Tracks is that, in this treatment, patient collaboration is not necessary because its basis is in selective wears and restoration with composite resin that acts continuously after construction. Additionally, it is low cost because sophisticated materials are not necessary for its execution and it is a simple technique that can be performed by general dentists, so it is not necessary to be a specialist in orthodontics [7, 8]. 

## 4. Conclusion

It can be concluded that Planas Direct Tracks were efficient in the correction of the posterior crossbite in the case report. If malocclusion is considered a public health problem, low-cost techniques that have easy execution are recommended so correction can be performed at an early age.
